# Outpatient Local Anaesthetic Transperineal Prostate Biopsy: A Four-Year Patient-Experience Audit (January 2021–January 2025)

**DOI:** 10.7759/cureus.93893

**Published:** 2025-10-05

**Authors:** Momen Sid Ahmed, David Dryhurst

**Affiliations:** 1 Department of Urology, King's College Hospital NHS Foundation Trust, London, GBR

**Keywords:** audit, local anaesthesia, outpatient, patient experience, transperineal prostate biopsy

## Abstract

Background: Transperineal prostate biopsy performed under local anaesthesia in the outpatient setting is increasingly being adopted. Patient-reported experience is central to service quality but is less frequently described than diagnostic or safety outcomes.

Objective: The objective of this study was to audit four years of patient-reported experience after outpatient local anaesthetic transperineal biopsy (LATP) in routine clinical practice.

Methods: We conducted a retrospective audit of consecutive patient feedback forms completed after LATP between January 2021 and January 2025 at a single centre. A five-item questionnaire captured overall positive experience, staff courtesy, clarity of communication, mention of significant pain during/immediately after biopsy, and free-text suggestions.

Results: A total of 471 feedback forms were analysed. Of these, overall positive experience was recorded in 268 (56.9%), staff courtesy in 227 (48.2%), and clear communication in 86 (18.3%). Mentions of significant pain occurred in 14 (3.0%). Waiting-time concerns were recorded in six (1.3%), dignity concerns in four (0.8%), and suggestions for improvement in 27 (5.7%). Year-specific counts of “significant pain” were 0/109 (0.0%) in 2021, 6/104 (5.8%) in 2022, 6/130 (4.6%) in 2023, 1/90 (1.1%) in 2024, and 1/38 (2.6%) in January 2025.

Conclusions: Across four years, clinic-based LATP yielded high proportions of positive experience indicators and a low frequency of patient-mentioned significant pain. Findings support the feasibility and acceptability of clinic-based LATP, with opportunities to further improve communication and flow.

## Introduction

Transperineal (TP) prostate biopsy has emerged as a preferred approach in many centres because it provides comparable diagnostic performance to the transrectal (TR) route while reducing infectious complications [1]. Contemporary guidance supports TP approaches, including performance under local anaesthesia in the outpatient clinic [2]. Clinic-based local anaesthetic TP biopsy (LATP) has demonstrated practicality and favourable tolerability in routine practice across multiple series [3-5].

Patient experience, spanning pain/discomfort, perceived dignity, and communication, is a critical quality metric [6,7]. The complications profile of prostate biopsy has been comprehensively reviewed [8]. Longstanding concerns about sepsis and antimicrobial resistance with the TR route further motivated the adoption of the TP approach [9]. Comparative evidence also shows that infection rates with TP biopsy remain very low, including in cohorts without routine prophylactic antibiotics [10,11]. Qualitative work has highlighted pre-biopsy anxiety, fear of pain, and embarrassment [7].

We implemented a simple, author-developed five-item patient-experience questionnaire for our outpatient LATP service. This audit describes four years of results and adds to the evolving literature on TP biopsy.

## Materials and methods

Design, setting, and participants

This was a single-centre, retrospective audit of consecutive patient feedback forms collected after outpatient LATP between January 1, 2021, and January 31, 2025. All male patients who underwent LATP during the period were invited to complete a brief feedback form before discharge; forms were anonymous and voluntary. The study was conducted at the Urology Outpatient Department (LATP clinic), Princess Royal University Hospital, Beckenham, London, United Kingdom.

Biopsy technique

All biopsies were performed transperineally under local anaesthesia in a dedicated outpatient room using real-time ultrasound guidance and a standardised freehand approach. Procedures followed the unit’s standard operating protocol for skin preparation, local anaesthetic infiltration, and systematic/targeted sampling as clinically indicated. No procedural images or proprietary content are reproduced in this manuscript.

Patient-experience instrument

Patient experience was captured using a five-item, author-developed checklist (see Appendices). Items recorded were: (i) overall positive experience, (ii) staff courtesy, (iii) clear communication, (iv) mention of significant pain during or immediately after biopsy (yes/no; with optional comment), and (v) free-text suggestions/concerns (including waiting time and dignity). "Significant pain” was recorded as a patient-judged binary item (Yes/No) with optional comment; no validated numeric pain scale (e.g., Visual Analogue Scale (VAS)/Numerical Rating Scale) was administered

Outcomes and analysis

The primary outcome was the proportion of forms recording an overall positive experience. Secondary outcomes included staff courtesy, clear communication, and mention of significant pain. Free-text comments were reviewed and categorised (e.g., waiting time, dignity). Data are presented descriptively as counts and percentages, n (%).

Ethics and governance

This work was registered locally as a clinical audit/service evaluation of routine care. Individual consent for anonymised, aggregate reporting of feedback was considered implicit in form completion. No patient-identifiable information was collected.

## Results

Overall patient-reported experience

A total of 471 feedback forms were received over the four years. Table 1 summarizes the aggregated results.

**Table 1:** Patient-reported experience after outpatient LATP (January 2021–January 2025) (N=471). LATP: local-anaesthetic transperineal biopsy

Item	Frequency (Percentage)
Overall positive experience	268 (56.9)
Staff courtesy and respect	227 (48.2)
Clear communication	86 (18.3)
Experience of significant pain during/immediately after biopsy	14 (3.0)
Waiting-time concerns (from free text)	6 (1.3)
Dignity/privacy concerns (from free text)	4 (0.8)
Suggestions for improvement (from free text)	27 (5.7)

Year-by-year counts, including pain mentions

Table 2 presents the total number of feedback forms filled in each year during the study period, and the number of respondents who mentioned significant post-procedure pain.

**Table 2:** Yearly distribution of feedback forms and mentions of significant pain.

Year	Feedback forms, n	Mentions of significant pain, n (%)
2021	109	0 (0.0)
2022	104	6 (5.8)
2023	130	6 (4.6)
2024	90	1 (1.1)
2025 (January)	38	1 (2.6)
Total	471	14 (3.0)

## Discussion

In this four-year audit of outpatient LATP, a majority of patients documented a positive overall experience (268/471, 56.9%) and staff courtesy (227/471, 48.2%). Only 3.0% (14/471) mentioned significant pain during or immediately after the procedure. These observations align with large real-world series reporting good tolerability of LATP under local anaesthesia [3] and with systematic reviews showing low procedural abandonment and generally modest pain scores for local-anaesthetic transperineal techniques [8].

Evidence consistently indicates that TP biopsy achieves a similar diagnostic yield to TR biopsy while reducing infectious events [1]. Contemporary guidance, technique reviews, and meta-analyses favour TP, noting markedly lower infection-related admissions and the potential to omit prophylactic antibiotics in selected settings [2,9-14]. Even where randomised trials report higher immediate pain/embarrassment with LATP versus TR under certain anaesthetic protocols, overall diagnostic advantages and infection-risk reduction still favour TP in most settings [14]. These nuances underscore the value of pre-procedure counselling and expectation setting.

Standardised outpatient LATP pathways have demonstrated practicality and performance across multiple centres [4,5,12,13]. Our service experience is congruent with those reports, suggesting that a consistent clinic pathway can yield favourable patient-experience metrics.

Strengths of this study include a four-year time frame and consecutive, routine clinic capture of feedback immediately post-procedure. Limitations of this study include the single-centre design, the use of a brief author-developed (non-validated) questionnaire, the lack of paired pain scales (e.g., VAS) or anxiolysis data, the absence of clinical covariates (e.g., prostate size, number of cores), and no TR comparator. Additionally, as feedback forms were returned voluntarily at discharge and the total number of LATP procedures performed during the audit period was not recorded, a response/capture rate could not be calculated; findings therefore describe respondents rather than all attendees. Nevertheless, the audit offers pragmatic insight into patient-perceived acceptability of LATP.

## Conclusions

Across four years and 471 feedback forms, outpatient LATP was associated with a high proportion of positive patient-experience indicators and a low frequency of patient-mentioned significant pain. These findings support the continued use and refinement of clinic-based LATP pathways.

Embedding brief pre-procedure counselling scripts, standardising local anaesthetic infiltration, and optimising clinic flow may further enhance patient experience. Incorporating validated patient-reported outcome measures and a numeric pain scale alongside time-to-discharge would provide a richer picture of acceptability and efficiency. Similar outpatient urology services can adopt this simple feedback approach to monitor and improve patient experience in real time.

## Appendices

Post-biopsy patient experience questionnaire (LATP-5)

Please complete before discharge. This form is anonymous and helps us improve the service.

**Table 3:** Patient-experience questionnaire used in this audit Notes: This five-item questionnaire was designed locally by the authors for service evaluation. It is not adapted from any third-party or copyrighted scale.

Item	Response options
1. Overall, was your experience today positive?	☐ Yes ☐ No ☐ Unsure
2. Were the nursing and clinic staff courteous and respectful?	☐ Yes ☐ No
3. Did the doctor explain the procedure clearly and answer your questions?	☐ Yes ☐ No
4. Did you experience significant pain during or immediately after the biopsy?	☐ Yes ☐ No
5. Any suggestions to improve your experience? (optional)	Free text: ________________________
